# Barriers to recruitment into emergency department-initiated palliative care: a sub-study of a multi-site, randomized controlled trial

**DOI:** 10.1186/s12904-021-00899-9

**Published:** 2022-02-15

**Authors:** Julia Brickey, Mara Flannery, Allison Cuthel, Jeanne Cho, Corita R. Grudzen, The EMPallA Investigators, Caroline Blaum, Lauren Southerland, Jason Bischof, Kei Ouchi, Marie-Carmelle Elie, Robert Swor, Karen Jubanyik, Keith S. Goldfeld, Susan E. Cohen, Arum Kim, Joseph Lowy, Jennifer S. Scherer, Nancy E. Bael, Ellin Gafford, Joshua Lakin, Paige Barker, Angela Chmielewski, Jennifer Kapo, Ada L. Rubin, Isabel Castro, Holden Caplan, Simar Kaur Randhawa, Jordan Carpenter, Gary Theroux, Rebecca Murray, Laura Stuecher, Nora Daut, Jennifer Bonito, Marie Bakitas, Romilla Batra, Juanita Booker-Vaughns, Garrett K. Chan, J. Nicholas Dionne-Odom, Patrick Dunn, Robert Galvin, Ernest A. Hopkins, Eric David Isaacs, Constance L. Kizzie-Gillet, Margaret M. Maguire, Neha Reddy Pidatala, Dawn Rosini, William K. Vaughan, Sally Welsh, Pluscedia G. Williams, Angela Young-Brinn, Martha Navarro

**Affiliations:** 1 University of North Carolina School of Medicine, Chapel Hill, NC USA; 2 Ronald O. Perelman Department of Emergency Medicine, New York University Grossman School of Medicine, Translational Research Building, 227 East 30th Street, Office 117, New York, NY 10016 USA

**Keywords:** Patient recruitment, Research subject recruitment, Patient selection, Patient participation, Patient engagement, Patient participation rates, Refusal to participate, Palliative care, Palliative supportive care, Palliative care medicine, Palliative medicine, Palliative nursing, Palliative care nursing, Hospice and palliative care nursing, Emergency department, Emergency care, Emergency medical services, Telehealth, Telephone, Telemedicine, Quality of life, Research design, Research strategy

## Abstract

**Background:**

Emergency department (ED) visits among older adults are common near the end of life. Palliative care has been shown to reduce ED visits and to increase quality of life among patients, but recruitment into these programs is often challenging. This descriptive analysis explores the barriers to enrolling seriously ill patients scheduled for discharge from the ED into palliative care research.

**Methods:**

This descriptive sub-study aims to assess the reasons why patients with advanced illness scheduled for discharge home from 11 EDs across the United States decline to participate in Emergency Medicine Palliative Care Access (EMPallA), a Phase IV randomized controlled trial (RCT) comparing two modes of palliative care delivery. Our aim was to understand why patients decline to enroll to improve future recruitment rates and expand care for patients discharged home from the ED. Research coordinators documented reasons that patients declined to enroll in the larger EMPallA trial; reasons for refusing participation were independently analyzed by two researchers to identify overarching themes.

**Results:**

Enrollment rate across all sites was 45%; of the 504 eligible patients who declined participation, 47% (*n* = 237) declined for reasons related to illness severity. 28% of refusals (*n* = 143) were related to the mode of palliative care delivery, while 24% (*n* = 123) were due to misconceptions or stigma related to palliative care. Less commonly, patients refused due to general research barriers (16.5%), family/caregiver barriers (11.7%), and physician-related barriers (< 1%).

**Conclusions:**

Patients with advanced illnesses presenting to the ED often refuse to participate in palliative care research due to the severity of their illness, the mode of care delivery, and misconceptions about palliative care. In contrast with other studies, our study found minimal physician gatekeeping, which may be the result of both changing attitudes toward palliative care and the nature of the ED setting. Robust training programs are crucial to overcome these misconceptions and to educate patients and providers about the role of palliative care. Future palliative care programs and study designs should recognize the burden this vulnerable population endures and consider alternative modes of care delivery in an effort to increase participation and enrollment.

**Clinical trials registration:**

NCT03325985, October 30, 2017.

## Background

Multiple studies have shown that palliative care improves quality of life in patients with advanced illness [1–3]. Patients receiving palliative care utilize fewer resources, have reduced Emergency Department (ED) visits and fewer hospital admissions, leading to reduced cost of medical care and higher satisfaction for patients and families [2, 4]. Nonetheless, engaging patients with advanced illness in palliative care and palliative care research is challenging for multiple reasons. The dearth of literature on this topic identifies significant barriers to enrolling patients in palliative care programs. LeBlanc and colleagues identify three major categories of barriers to palliative care research: 1) patient issues, 2) gatekeeping, and 3) ethical issues [5]. Patients who are suitable for palliative care often feel too sick or burdened by their illness to participate, and limited life-expectancy among this population complicates data collection [6, 7]. Gatekeeping is common among this population and occurs when a physician, family member, or caregiver prevents a patient from participating in research. Moreover, conducting research among such a vulnerable population raises ethical concerns, which although valid, can be addressed [8]. Other barriers cited in the literature include difficulties in communication regarding end-of-life care and lack of training in palliative care amongst providers [9–12].

Despite these barriers, the limited research that does exist highlights the overwhelmingly positive benefits of palliative care in patients with advanced illnesses [1, 2, 13]. Furthermore, recent studies point to the ED as a potential setting in which to enroll patients in palliative care programs, as ED visits are extremely common among patients with advanced illness [14]. Recruiting patients into palliative care research in the ED setting may pose unique challenges due to the fast-paced environment, lack of an ongoing relationship with the patient, and high symptom burden that brought patients there to begin with [15, 16]. Despite this limited research, few studies if any have attempted to understand the barriers to recruiting patients into palliative care within an ED setting. Thus, additional research is needed to understand how best to recruit this vulnerable population from the ED, engage them in research and optimize methods of palliative care delivery.

The parent study, Emergency Medicine Palliative Care Access (EMPallA), is a multi-site randomized controlled trial (RCT) designed to compare the effectiveness of two modes of community-based palliative care delivery for patients with advanced illness. Patients are recruited into EMPallA from the ED or observation unit and randomized to either nurse-led telephonic case management or outpatient specialty palliative care. The aim of this paper is to clarify the barriers to enrolling seriously ill patients scheduled for discharge from the ED into palliative care research.

## Methods

This descriptive sub-study aims to assess the reasons why patients with advanced illness scheduled for discharge home from 11 EDs across the United States decline to participate in EMPallA, a Phase IV randomized controlled trial (RCT) comparing two modes of palliative care. Specifically, the patients who fulfill eligibility criteria but choose not to consent to participate in the EMPallA study will be the focus of this paper, as this group of patients provides valuable information to improve recruitment in future trials.

The EMPallA peer-reviewed trial leveraged the Clinical Trials Transformation Initiative (CTTI) framework prior to active recruitment in order to maximize recruitment strategies and best practices [17]. Eligibility criteria for the EMPallA study include patients 50 years of age or older who presented to the ED with a qualifying illness (defined as advanced cancer or end-stage organ failure) and were scheduled for ED discharge or observation status. Patients were also required to have health insurance, speak English or Spanish, and reside within the geographic area. Patients were not eligible if they had previously received hospice or palliative care, were admitted to inpatient services, had dementia, or lived in a skilled nursing facility (SNF) or similar assisted living facility (ALF). Additional study details can be found in the published protocol paper [18].

Research coordinators (RCs) at each ED received extensive training prior to active patient approach in the ED; trainings leveraged stakeholder feedback from palliative care physicians, as well as the EMPallA Study Advisory Committee (SAC) patient stakeholder group, to ensure proper messaging as per the CTTI recruitment communication planning recommendation [17]. Additionally, RCs had the opportunity to practice the script and pitch with SAC members via teleconference to obtain feedback and suggestions for improvement prior to approaching eligible patients.

**Fig. 1:**
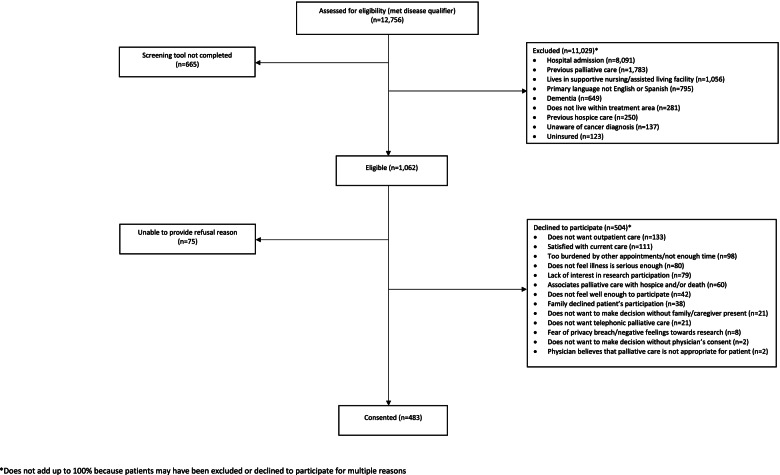
CONSORT Diagram

Patients were screened through the electronic health record. If they did not meet one or more of the illness qualifiers they could not be approached and their ineligibility reasons were documented in REDCap by the RC (Fig. 1). Eligible patients who were approached at bedside but declined participation in EMPallA were asked to provide a reason for their refusal which was documented for the sub-study in REDCap, a secure, web-based application that served as the central research database [19]. Patients were permitted to provide more than one reason, and patient refusals were not mutually exclusive; each reason for refusal was accounted for in the analysis. Initially, reasons for refusal were recorded in a ‘free text’ manner, extracted and summarized from REDCap by an NYU data analyst, and reviewed on a monthly basis by two researchers. Like reasons were grouped and eventually compiled into a checklist in REDCap in December 2018. The checklist response choices were continuously refined in REDCap based on emerging themes from an ‘other’ free-text category. For this manuscript, reasons were independently analyzed by two researchers to identify themes, and discussed in-depth until consensus was reached.

Weekly team meetings of RCs from all 11 sites occurred as an opportunity to discuss each refusal reason and strategize potential solutions in an effort to maximize recruitment. In order to monitor eligibility and enrollment, data was organized into a Consolidated Standards for Reporting of Trials (CONSORT) diagram (Fig. 1) [20–22]. All data represented in this manuscript is from the reporting period (March 28, 2018 - January 31, 2020). Based on data from the Principal Investigator’s preliminary RCT, which informed the planning of the EMPallA RCT and enrolled eligible patients at a rate of 64%, we expected a recruitment rate of 50% [23–25].

## Results

The CONSORT diagram (Fig. 1) depicts the process of patient recruitment in the larger EMPallA study from screening to consent. As of January 31, 2020, 12,756 patients with a qualifying illness have been identified, of which 1062 were deemed eligible for EMPallA (8%). The majority of patients screened (11,029) were not eligible and could not be approached because they met one or more exclusion criteria (Fig. 1). Reasons for ineligibility based on exclusion criteria are included in Fig. 1, with admission to the hospital being the main reason why patients were not approached. The reasons for ineligibility will not be further discussed in this paper as they are specific to EMPallA and thus not generalizable to other study designs; the scope of this paper comprises patients who meet eligibility. There was a subset of patients (665) for whom the screening tool was not completed because RCs were unable to approach them prior to discharge. Of the 1062 eligible patients, 483 were successfully enrolled, and 579 were not enrolled, yielding an overall enrollment rate of 45%. Enrollment rates varied widely across sites (ranging from 8 to 92%). Notably, of the 579 patients who were eligible but not enrolled in EMPallA, 75 patients were unable to provide reasons for refusal. This was frequently a result of either insufficient documentation by RCs, or patients not feeling well enough to engage in conversation with the RCs to provide thorough rationale. Thus, 504 patients provided reason(s) for their refusal to participate in EMPallA, and this subset of patients are the focus of this paper.

Table 1 outlines the frequency of each refusal by category. Results are based on the number of patients that cited each reason, and patients were allowed to provide more than one reason. Of the 504 eligible patients who refused to participate, 237 (47.0%) refused due to “barriers related to illness severity.” Of these patients, 98 (19.4%) stated that they were too burdened by other appointments or did not have enough time to participate. One-hundred eleven patients (22.0%) said that they were satisfied with their current care, often stating, “I’m happy with the care that I’m getting” or “I have everything I need.” A smaller proportion of patients (42 [8.3%]) did not feel well enough to participate. Patients also commonly refused due to “misconceptions/stigma related to palliative care” (123 [24.4%]). Of these 123 patients, more than half did not feel that their illness was serious enough to need palliative care (80 [15.9%]). The remainder of patients refused because they associated palliative care with hospice and/or death (60 [11.9%]), making statements such as: “I’m not dying” and “I’m not there yet.” One-hundred forty-three patients (28.4%) refused due to the mode of palliative care delivery. The overwhelming majority of these patients (133 [26.4%]) did not want to or could not commit to attending the outpatient clinic visits. In contrast, only 21 patients (4.2%) stated that they did not want to receive telephonic palliative care. In some instances, patients wanted the choice to be placed in one intervention arm over another but refused study participation since they could not be guaranteed placement into their preferred treatment arm.

**Table 1:** Reasons that eligible patients refused to participate (*n* = 504)

Reason for refusal	Patients, no. (%)
**Barriers related to illness severity**	**237 (47.0)**
➢ Too burdened by other appointments/not enough time	98 (19.4)
➢ Satisfied with current care	111 (22.0)
➢ Doesn’t feel well enough to participate	42 (8.3)
**Misconceptions/stigma related to palliative care**	**123 (24.4)**
➢ Does not feel illness is serious enough	80 (15.9)
➢ Associates palliative care with hospice and/or death	60 (11.9)
**Mode of palliative care delivery**	**143 (28.4)**
➢ Does not want outpatient palliative care	133 (26.4)
➢ Does not want telephonic palliative care	21 (4.2)
**General research barriers**	**83 (16.5)**
➢ Lack of interest in research participation	79 (15.7)
➢ Fear of privacy breach/negative feelings towards research	8 (1.6)
**Family/caregiver barriers**	**59 (11.7)**
➢ Family declined patient’s participation	38 (7.5)
➢ Does not want to make decision without family/caregiver present	21 (4.2)
**Physician-related barriers**	**4 (0.1)**
➢ Does not want to make decision without physician’s consent	2 (.04)
➢ Physician believes that palliative care is not appropriate for patient	2 (.04)

*Note*: categories and subcategories may not add up to 100% because patients may have declined participation for multiple reasons

Eighty-three patients (16.5%) refused to participate for reasons categorized as “general research barriers.” Patients typically exhibited a lack of interest in research participation (79 [15.7%]). However, some patients reported a fear of privacy breach or had negative feelings towards research (8 [1.6%]). A small proportion of patients did not participate due to family or caregiver barriers (59 [11.7%]), and in some cases, a family member or caregiver recommended the patient not participate (38 [7.5%]). Twenty-one (4.2%) reported that they did not want to make a decision without a family member or a caregiver present. Only four patients out of 504 (0.1%) refused because of physician-related barriers. Two patients preferred not to make a decision without consent from their physician, and there were two instances in which the physician declined on behalf of the patient.

## Discussion

### Main findings

The most common patient refusal reasons were barriers related to 1) the severity of the patient’s illness, 2) misconceptions or stigma related to palliative care, and 3) the mode of palliative care delivery. Barriers including illness severity, misconceptions and family/caregiver and physician gatekeeping are previously cited in the literature, but the mode of palliative care delivery is a finding that is unique to this study. We hypothesized that family/caregiver and physician gatekeeping would be a more prevalent refusal reason and barrier, but we found these to be far less common in our study. Thus, our findings provide insights into ways in which researchers can tailor recruitment strategies as well as design palliative care programs which meet the needs of this patient population.

Aligning with lessons learned from the Principal Investigator’s prior randomized controlled trial of Palliative Care in the ED, our enrollment goal was 50% and overall enrollment rate was 45% across the 11 sites [23–25]. Despite being slightly under our target goal, the data we have collected thus far provide valuable lessons learned for future research in this field. As it is well documented that research within palliative care populations is difficult, this enrollment rate is not particularly surprising [12, 23, 26]. The FamCope Trial, which aimed to test the feasibility of nurse-led, family-coping-oriented palliative home care in patients with advanced cancer, encountered higher refusal rates in comparison to our study (66% vs. 55%) but cited similar reasons for refusal such as a “lack of energy” or being “too sick.” [9] In contrast to the FamCope Trial, our study found that patients often declined to participate because they felt that they were satisfied with their current care. Although this is not a frequently reported barrier in the literature, other studies also have reported that patients with advanced cancer often refuse to participate in palliative research due to satisfaction with their current care [16]. Presumably, there are some patients with advanced illness whose needs are being met with their current care, and thus do not feel as though they would benefit from palliative care.

Although other studies have cited physician gatekeeping as a major recruitment barrier, we found this to be a negligible barrier [11, 23]. The reasons for this are undoubtedly multifactorial, but it is possible that this reflects a shift of attitude toward palliative care within the healthcare community. While some research suggests that providers still hold perceptions that palliative care is only appropriate at the end of life, there is a growing body of evidence which demonstrates that physicians have a greater understanding of the role of palliative care and are more confident in referring patients to palliative care services [12, 27, 28]. A recent study analyzing the perceptions of palliative care among healthcare providers before and after implementation of a palliative medicine division found increased attendance in educational activities and increased confidence in palliative care [28]. Within the same study, providers who favored co-management with palliative care held core values that aligned with current concepts, such as advanced care planning [28]. We expect that as the number of palliative care programs in hospitals across the country increases, understanding and acceptance of palliative care amongst providers will continue to grow and foster a new set of beliefs and norms [29].

Additionally, we believe that recruiting patients from the ED helped us to overcome some of the challenges faced by other studies, particularly in regard to gatekeeping. Notably, the ED physicians in our study were given the opportunity to opt-out their patients in the EMPallA study prior to recruitment, but few took the study team up on the offer. Kars et al. report on patterns of gatekeeping in palliative care research, noting that gatekeeping is motivated by protection of patients who are deemed to be vulnerable [30]. Due to the nature of Emergency Medicine whereby physicians typically do not have long-standing relationships with their patients, we hypothesize that they may be less likely to prevent patients from enrolling in palliative care, as they may not feel comfortable making such a care decision. This is further supported by the fact that most of the studies which cite physician gatekeeping as a barrier to palliative care recruitment attempted to recruit patients in the outpatient setting.

Moreover, it is well known that ED visits are common among patients near the end of their life; thus, the ED represents a central location which may be appropriate for recruitment into palliative care research. In a paper focused on interviewing principal investigators and clinical research coordinators regarding their experience with a palliative care clinical trial, Hanson and colleagues highlight the difficulties associated with recruiting patients from multiple healthcare settings [31]. By utilizing the ED as the central recruitment site, the need for recruitment from outpatient clinics or long-term care facilities can be essentially eliminated, and in turn, the recruitment process simplified. Our results also demonstrated that family and caregivers did not impede recruitment. RCs anecdotally expressed many patients were often alone in the ED when approached, which may partially explain why family and caregiver gatekeeping did not hinder recruitment in this study. More detailed data collection is needed to understand this concept more deeply.

This study also further demonstrates that the mode of palliative care delivery often factored into a patient’s decision to participate in the study. Of the patients who did not participate due to mode of palliative care delivery, the majority indicated that they did not want to be randomized into the outpatient palliative care arm, due to inability or unwillingness to make it to in-person clinic visits. In contrast, relatively few patients said that they did not want to receive telephonic care. This finding is noteworthy for future researchers and healthcare system leadership as it demonstrates that patients may be more open to receiving palliative care if they do not have to attend in-person outpatient clinic visits. To our knowledge, these are new findings which have not been reported in other studies. Aligning with our results, these findings suggest that patients with advanced illness may be reluctant to add an additional in-person clinic appointment to their schedules, as they may be overburdened with multiple appointments. Telemedicine visits (e.g. phone call, video chat) may be a feasible alternative to in-person clinic visits, and other studies have demonstrated success of telephonic palliative care [32, 33]. Future studies should continue to explore the effectiveness of telehealth visits as an alternative mode of care delivery for this specific population.

### Implications for future studies

Future studies which aim to engage patients in palliative care research must recognize that this population is inherently difficult to recruit given the extent of their illness and symptoms. To that end, researchers must anticipate barriers related to illness severity and attempt to minimize other variables as much as possible. We suggest implementing a robust training infrastructure and documentation system for RCs. Creating a systematic and standardized approach can minimize bias and may increase fidelity of RCs. For those patients who express not having enough time to attend another appointment, knowing the details of the outpatient clinic (clinic location, ability to schedule appointments on the same day as other appointments) is essential. Furthermore, we advise meeting with the site outpatient team to discuss how they would navigate this reason for refusal as early as possible in the study trajectory. Early engagement with a palliative care provider is also necessary, as they can make recommendations on navigating the conversation when a patient expresses they are satisfied with their care but may have never received palliative care. A ‘decision tree’ workflow for how to address a patient feeling too ill to enroll (for example, if the team member could offer to re-approach at a later time), may also help increase enrollment. Sharing robust ‘tip sheets’ based on these conversations would improve fidelity across study teams. Training should include CTTI recruitment communication planning such as: developing a standard patient-centered script, shadowing senior RCs, supervised patient recruitment by senior RCs, proper documentation of each encounter, and ongoing supervision and oversight of all RCs by either the site project manager or principal investigator.

Prior to active recruitment, we recommend RCs role-play patient recruitment scenarios with patient stakeholder groups like the SAC in order to receive constructive feedback. The training infrastructure should be closely monitored. To minimize site variations in the context of our multi-site study, we conducted in-person site visits prior to launch, developed standard materials that could be personalized with site specific logos, and implemented weekly calls to discuss recruitment barriers and facilitators. Calls were also used to brainstorm strategies in overcoming recruitment barriers. All facilitators were disseminated to all sites enrolled in the study. As the NYU Research Team closely monitored and evaluated recruitment metrics as per the CTTI framework recommendation, high performing recruitment sites were paired with low performing sites to provide feedback and support.

More research is needed on how best to engage physicians to ensure they are allies, rather than barriers, in the recruitment process. We suggest providing physicians with the autonomy to exclude their patients, as we did within this study. Another suggestion could include engaging physician stakeholders during the development of the research project, as they likely hold unique perspectives on the best ways to recruit patients.

Furthermore, our findings indicate that future studies will likely encounter difficulties secondary to misconceptions of palliative care. Many patients we encountered either misunderstood the role of palliative care or equated it with hospice care, a barrier that has reported in other studies as well [9, 16].

To mitigate this, we suggest an approach centered on patient education and messaging consistency. The recruitment script should clearly explain the role of palliative care in patient-friendly language. For example, it should be emphasized that palliative care is intended to add a layer of support and can be delivered in conjunction with life-prolonging treatment. As previously mentioned, RCs should be given ample opportunity to practice the script to ensure consistency and comfortability and this practice should be revisited periodically. Involving a palliative care physician in the training process is crucial, as they can provide important feedback regarding proper language and messaging techniques.

Many of the aforementioned strategies align with recommendations made by LeBlanc and colleagues [5]. They suggest employing the principles of social marketing to create a protocol aimed at improving recruitment into palliative care research, which helped them to achieve a recruitment rate > 75%. Their study cites the importance of involving stakeholders, role-play training, standardization of wording, and messaging based on known barriers to palliative care research, all of which have been a focus of our study as well. They also incorporated what they refer to as a triage algorithm, in which non-study personnel gauged patients’ interest prior to having a research nurse approach them. Not only did this save time, but it also allowed for better identification of possible participants, and future studies may want to consider employing a similar method.

### Strengths/limitations

The major strength of this paper is the generalizability of the results across different geographic ED contexts (11 unique EDs across the country), thus demonstrating that it is feasible to recruit patients who present to the ED with multiple disease etiologies into palliative care research studies. Understanding patient barriers within this sub-population is integral in order to plan and develop trials that leverage successful recruitment strategies. While other palliative care studies are limited to patients with advanced cancer, our study recruited patients with advanced cancer, congestive heart failure, chronic kidney disease, and chronic obstructive pulmonary disease. By including these patients, we have captured a wider scope of patients with advanced illness and improved the generalizability of these findings. Furthermore, it is relatively uncommon for ED studies and studies in general to record reasons for refusal. In the context of such a large study, these refusal reasons provide unique insight into the reasons why patients with advanced illnesses are hesitant to engage in palliative care research while presenting to the ED. On that premise, we could have strengthened this study by employing qualitative interviews with patients who refused to participate. This would have provided us with more in-depth information regarding patients’ reasons for refusal and areas for improvement to increase enrollment.

Our CONSORT diagram reveals that a significant portion of patients were deemed ineligible due to hospital admission. This posed a unique limitation to EMPallA, as we were unable to collect refusal data from patients admitted into the hospital. Nonetheless, this exclusion criterion was essential for our study design in order to target a specific group of patients who may not otherwise have access to palliative care programs. Notably, palliative care services are available to admitted patients, but few resources exist for patients who are discharged home from the ED [26, 34, 35].

Furthermore, our study identified that family and caregiver gatekeeping was not a common barrier to enrollment in our patient population, but we are unable to draw definitive conclusions due to limited data collection specific to this refusal reason. In research, it is challenging to capture information and the rationale of non-participants; thus, future studies should try to incorporate qualitative methods such as content analyses in order to thoroughly interpret findings.

## Conclusion

By providing greater insight into why patients with advanced illness refuse to participate in palliative care research, we have been able to explore the ways in which we can successfully engage this patient population. In particular, misconceptions related to palliative care may prevent patients from enrolling in palliative care programs, so it is essential that patients have a clear understanding of the role of palliative care. This requires a strong training infrastructure for RCs. Furthermore, engagement with palliative care physicians and ongoing communication across recruitment sites are essential in order to overcome enrollment challenges. Future studies with palliative care populations must design programs that meet the needs of this population, which may include some form of telemedicine. Although it is often difficult to engage patients with advanced illness in palliative care research, our study demonstrates that it is both feasible and imperative that we continue efforts to engage with this patient population.

## Data Availability

All data generated and/or analysed during the current study or sub-study are available from the corresponding author on reasonable request.

## Abbreviations

ALF: Assisted Living Facility
CONSORT: Consolidated Standards for Reporting of Trials
CTTI: Clinical Trials Transformation Initiative
ED: Emergency Department
EMPallA: Emergency Medicine Palliative Care Access
RCT: Randomized Controlled Trial
RC: Research Coordinator
REDCap: Research Electronic Data Capture
SAC: Study Advisory Committee
SNF: Skilled Nursing Facility
